# Retraction: Proteasome storage granules protect proteasomes from autophagic degradation upon carbon starvation

**DOI:** 10.7554/eLife.82988

**Published:** 2022-09-02

**Authors:** Richard David Vierstra

**Keywords:** *A. thaliana*, *S. cerevisiae*

Marshall RS, Vierstra RD. 2018. Proteasome storage granules protect 26S proteasomes from autophagic clearance during carbon starvation. *eLife* **7**:e34532. doi: 10.7554/eLife.34532.

As the corresponding author I retract the eLife paper cited above based on unexplained irregularities associated with a collection of western blots and inappropriate placement of several confocal fluorescence micrographs generated by the first author Dr. Richard S. Marshall who was responsible for all the experiments and the first preparation and final drafts of the figures. As first identified by reports on PubPeer, I have thoroughly investigated the allegations and confirmed both the duplication of several micrographs and duplicated horizontal narrow bands in the background regions of a number of western blots, and further discovered poor archiving of the experimental data and associated yeast strains. The modifications of the western blots first flagged by PubPeer were particularly perplexing from my analysis of the original x-ray films of the blots as they appear as replicated narrow slices across the blots positioned often between the GFP fusion and free GFP released by autophagic turnover. These bands impacted only background regions and did not appear to be designed to cover any extraneous bands nor to splice two different western blot images together. Dr. Marshall has not yet offered an explanation for these duplications. While the main premise of the eLife report, i.e. to demonstrate a role of proteasome stress granules in protecting proteasomes from autophagic turnover during carbon and/or energy starvation, appears to remain intact from my analysis of the data and subsequent independent studies by us and others related to key aspects of the study, I am not sure that all aspects of the studies therein are valid. We are currently repeating several parts of the paper and hope to provide appropriate and rigorous confirmatory data in the near future. Consequently, I feel that it is prudent at this time to retract the paper to avoid confusion in the literature. I apologize to the readers for these errors/irregularities. I, as the last author, was responsible for ensuring scientific integrity for this publication but clearly failed as a ‘gatekeeper’ in this case. The corresponding author Richard D. Vierstra agrees to this retraction. The following author was also contacted but he did not provided comments nor explanations for the errors: Richard S. Marshall.

